# Anti-inflammatory and anti-arthritic effects of yucca schidigera: A review

**DOI:** 10.1186/1476-9255-3-6

**Published:** 2006-03-29

**Authors:** PR Cheeke, S Piacente, W Oleszek

**Affiliations:** 1 Department of Animal Sciences, Oregon State University, Corvallis, OR 97333, USA; 2 Desert King International, 7024 Manya Circle, San Diego, CA 92154, USA; 3 Department of Pharmaceutical Sciences, University of Salerno, via Ponte Don Melillo-84084, Fisciano, Salerno, Italy; 4 Department of Biochemistry, Institute of Soil Science and Plant Cultivation, ul. Czartoryskich 8, 24100 Pulawy, Poland

## Abstract

*Yucca schidigera *is a medicinal plant native to Mexico. According to folk medicine, yucca extracts have anti-arthritic and anti-inflammatory effects. The plant contains several physiologically active phytochemicals. It is a rich source of steroidal saponins, and is used commercially as a saponin source. Saponins have diverse biological effects, including anti-protozoal activity. It has been postulated that saponins may have anti-arthritic properties by suppressing intestinal protozoa which may have a role in joint inflammation. Yucca is also a rich source of polyphenolics, including resveratrol and a number of other stilbenes (yuccaols A, B, C, D and E). These phenolics have anti-inflammatory activity. They are inhibitors of the nuclear transcription factor NFkappaB. NFkB stimulates synthesis of inducible nitric oxide synthase (iNOS), which causes formation of the inflammatory agent nitric oxide. Yucca phenolics are also anti-oxidants and free-radical scavengers, which may aid in suppressing reactive oxygen species that stimulate inflammatory responses. Based on these findings, further studies on the anti-arthritic effects of *Yucca schidigera *are warranted.

## Introduction

*Yucca schidigera *is an herbaceous plant of the lily family, native to the deserts of the south-western United States and northern Mexico. This plant was used in traditional medicine by Native Americans to treat a variety of ailments including arthritis. Yucca products are currently used in a number of applications. Yucca powder and yucca extract are used as animal feed additives, as discussed in detail by Cheeke and Otero [1]. Beneficial effects in livestock and poultry production include: increased growth rate and improved feed conversion efficiency, reduction in atmospheric ammonia in confinement animal and poultry facilities, anti-protozoal and nematocidal activity, modification of ruminal microbe populations, inhibition of Gram-positive bacteria, reductions in stillbirths in swine, reduction in egg and tissue cholesterol contents, and anti-arthritic activity in horses and dogs. Other applications include the use of yucca extract as a foaming agent in beverages, and use in crop production as nematode and fungi-control agents, as a soil wetting agent, and crop growth stimulant. Yucca products have GRAS status, so are FDA-approved for use in humans.

## Yucca saponins

**Figure 1:**
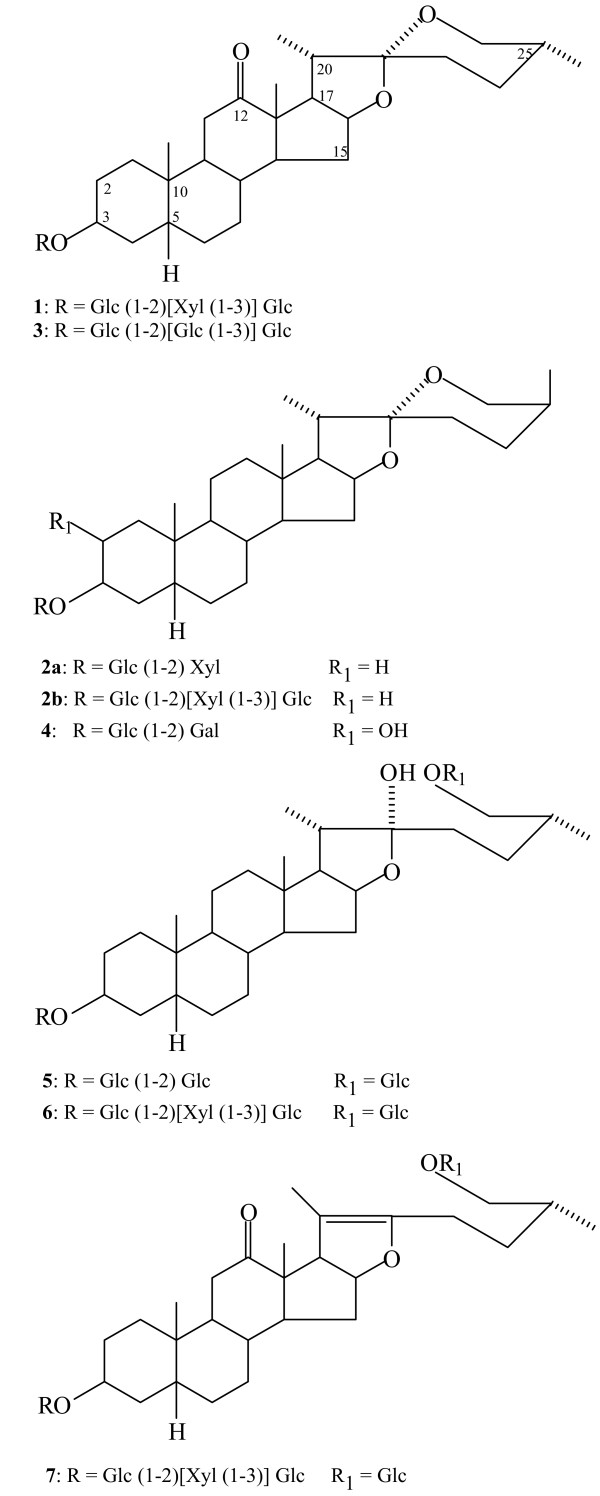
Chemical structures of saponins identified in *Yucca schidigera *bark [17]. Compounds 1–4 represent monodesmosidic and 6–7 bidesmosidic structures.

Yucca contains a number of phytochemicals which contribute to these effects. The best known are the steroidal saponins. Saponins are natural detergents [2] that form stable foams. Saponins contain a lipophilic nucleus (the sapogenin) and one or more side chains of hydrophilic carbohydrate (Fig. 1). Thus the intact saponin molecule is a surfactant, with both fat-soluble and water-soluble moities. It has been known for many years [3] that saponins form insoluble complexes with cholesterol. The hydrophobic portion of the saponin (the aglycone or sapongenin) associates (lipophilic bonding) with the hydrophobic sterol nucleus of cholesterol in a stacked micellar aggregation [4].

Interactions of saponins with cholesterol and other sterols account for many of their biological effects, particularly those involving membrane activity. It was demonstrated more than 45 years ago that dietary saponin reduces blood cholesterol levels [5,6]. This effect is a result of the saponins binding to cholesterol excreted in bile, thus inhibiting entero-hepatic cholesterol recycling. Dietary yucca extracts lower total and LDL cholesterol levels in hypercholesterolemic humans [7]. Saponins affect the permeability of intestinal cells by forming complexes with cholesterol in mucosal cell membranes [8]. In a similar manner, saponins have anti-protozoal activity by complexing with cholesterol in protozoal cell membranes, causing damage to the integrity of the membrane, and cell lysis. This has been well demonstrated with rumen protozoa *in vivo *[9-11]; and *in vitro *[12,13]. The antiprotozoal (cholesterol-binding) activity requires the intact saponin structure with both nucleus and side chain present.

Protozoal diseases in which part of the life cycle occurs in the gastrointestinal tract respond to the anti-protozoal activity of saponins. For example, yucca saponins are as effective as the drug metronidazole in killing tropozoites of *Giardia lamblia *in the intestine [14]. *Yucca schidigera *contains as much as 10% of steroidal saponins in its stem dry matter, making this plant one of the richest commercial sources of saponins. Acid hydrolysed fractions of yucca contain both furostanol and spirostanol aglycones. These include sarsapogenin, markogenin, smilagenin, samogenin, gitogenin and neogitogenin [15]. In the plant they can be found in a multi-component mixture of glycosides [16,17]. They can be found both as monodesmosides with one sugar chain attached at 3-*O*- and bidesmosides with two sugar chains at 3-*O*- and 26-*O*- positions (Fig. 1). Tanaka and co-workers identified as many as 13 structurally different saponins, but all of them were monodesmosides, given trivial names YS-I-XIII [16]. In the work of Oleszek and co-workers, eight individual saponins were isolated and identified out of which five were known spirostanol and three new furostanol structures [17]. However, monodesmosides made up about 93% of total saponins present.

## Yucca phenolics

**Figure 2:**
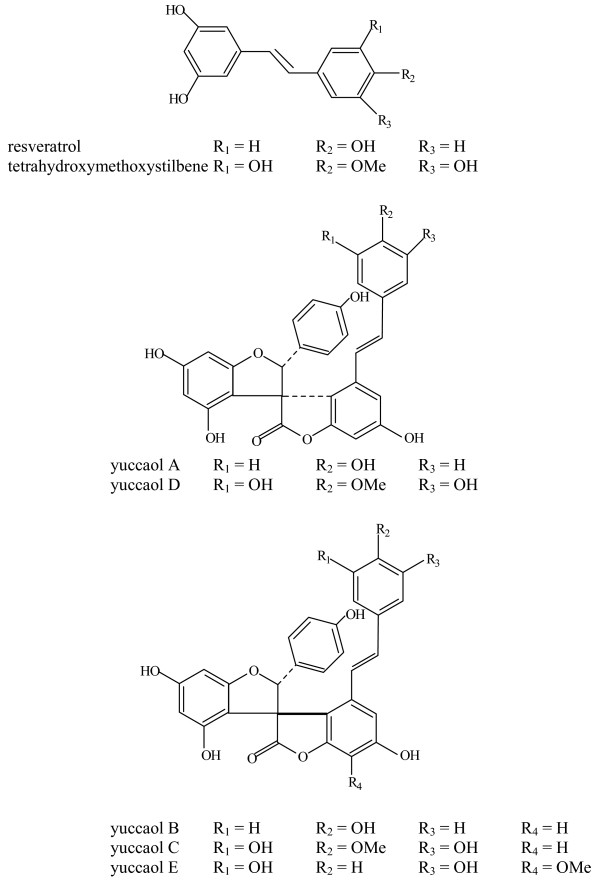
Structures of yucca phenolics.

Recently it has been recognized that yucca contains other physiologically-active constituents, particularly polyphenols. Two stilbenes, including trans-3,3',5,5'-tetrahydroxy-4'-methoxystilbene and trans-3,4',5-tetraxydroxystilbene (resveratrol) were identified in yucca bark. Also, some unique compounds with *spiro *confirmation were isolated and characterized. These included the spirobiflavonoid, larixinol, biosynthesized by combining two C_15 _units of flavonoid origin, previously identified in *Larix gmelini *and a number of novel spirostructures, which were given trivial names of yuccaols A-E [18,19] (Fig. 2). These compounds are composed of a C_15 _unit probably originating from the flavonoid skeleton and a C_14 _stilbenic compound linked via γ-lactone ring. Resveratrol makes up the stilbenic portion of yuccaols A and B and trans-3,3',5,5'-tetrahydroxy-4'-methoxystilbene is the stilbene in yuccaols C, D and E. By the analogy to the biosynthesis of larixinol it was presumed that most probably these compounds are synthesized by the attachment of the stilbenic derivative to the carbocationic intermediate occurring during the oxidation of flavanone to flavanol and subsequent rearrangement of this intermediate. Resveratrol was identified previously in grapes and is believed to be a phytoalexin produced by the plant to fight fungal colonization [20]. In yucca, this compound as well as its methoxyderivative and yuccaols can be found exclusively in yucca bark (Table 1), which is a dead tissue; it is not clear how these compounds are accumulated in this plant organ. Since yucca bark is a component of commercially available yucca powder, these compounds are present exclusively in this product; they are not present in yucca extract obtained by mechanical extraction.

**Table 1:** Concentrations of phenolics in different fractions of yucca [28].

**Item**	**Resveratrol (mg/g)**	**Yuccaols (mg/g)**
**Yucca bark**	21.7	72.6
**Yucca whole plant powder**	3.2	10.0
**Yucca extract**	NP*	NP

*NP = not present.

The chemistry and bioactivity of yucca saponins and phenolics have recently been reviewed by Piacente et al. [21].

## Anti-arthritic effects of yucca

Yucca products have been used for many years for reputed anti-arthritic effects, both by Native Americans and more recently by the nutraceutical industry. Whole yucca plant powder in tablet form is a common nutraceutical. The only direct studies of anti-arthritic effects of yucca are those of Bingham [22-24], who reported that symptoms of pain and swelling in arthritic human patients were relieved by consumption of yucca tablets. Bingham's work was reported in an obscure journal, and has apparently not been recognized as valid by the arthritis research community. Nevertheless, Bingham's reports have led to the widespread use of yucca products for treatment and prevention of arthritis not only in humans but also in horses and dogs.

Bingham [22] proposed that yucca saponins have anti-protozoal activity, which suppresses protozoal infection of the intestine. Bingham [22] reported that R. Wyburn-Mason had observed a free-living protozoan, *Naegleria*, universally present in the joints of arthritic patients [25]. Tropozoites of the organism reportedly were found in the intestine. Support for this theory was provided by the effectiveness of metronidazole, an anti-protozoal drug, in arthritis treatment. Saponins are also effective anti-protozoal agents. Yucca saponins are as effective as metronidazole in killing giardia tropozoites in the intestine [14]. Thus, if the protozoal theory of causation of arthritis has any merit, a role of yucca in arthritis treatment can be advanced on the basis of the anti-protozoal activity of yucca saponins.

There are well-known interactions between rheumatoid arthritis, chronic inflammatory disease, and food and nutrition [26,27]. Of particular importance are nutrients that stimulate the formation of oxidants and peroxides (e.g. unsaturated fatty acids, iron), which promote inflammatory disease, and antioxidants (e.g. vitamin E) and omega-3 fatty acids, which protect against auto-oxidation. Yucca compounds may have roles in these effects. Yucca polyphenols are potent antioxidants [18,21,28]. Yucca saponins are known to reduce iron absorption [29] and may reduce fatty acid absorption by sequestering bile acids necessary for micelle formation and fat absorption [4].

Cordain [30] stated, "Despite the almost universal clinical observation that inflammation of the gut is frequently associated with inflammation of the joints and vice-versa, the nature of the relationship remains elusive." These authors reported that arthritis is associated with intestinal bacterial overgrowth of *Escherichia coli *and *Lactobacillus lactis*. Yucca saponins have antibacterial properties [31,32], although *Lactobacillus *spp. and *E. coli *may be tolerant of yucca extract and yucca saponins [31]. Thus, a beneficial effect of yucca on arthritis could involve anti-protozoal, anti-oxidant and anti-bacterial activities. As previously mentioned, the drug metronidazole attenuates gastrointestinal inflammation and can prevent activation of arthritis in animal models [30]. Yucca saponins are as effective as metronidazole in control of intestinal protozoa [14].

**Figure 3:**
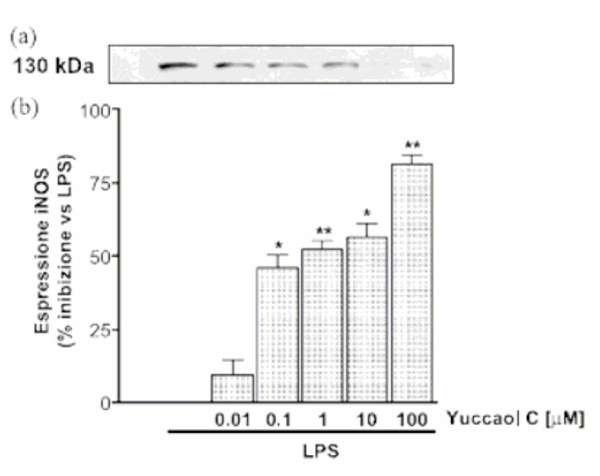
Representative blot of iNOS expression (a). Densitometric analysis of concentration-dependent effect of Yuccaol C (0.01–100 μM) on LPS-induced iNOS expression in J774.A1 macrophages (b). Yuccaol C was added 1 h before and simultaneously with LPS challenge. Values, mean ± s.e.m., are expressed as %inhibition of at least 6–9 independent experiments with 3 replicates each. Comparisons were made using one way ANOVA test. *P < 0.05, and **P < 0.01 [34]

**Figure 4:**
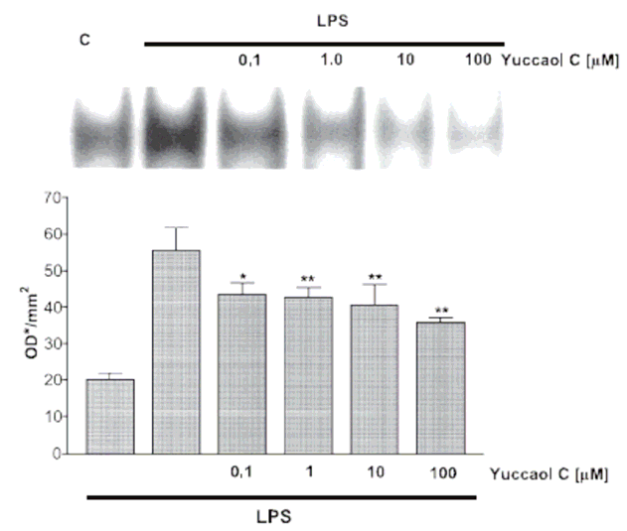
Effect of yuccaol C (0.01–100 μM) on NF-kB in LPS-stimulated J774.A1 macrophages. Values, mean ± s.e.m., are expressed as optical density/mm^2 ^of at least 3 independent experiments with 3 replicates each. Comparisons were performed using one way ANOVA test. *P < 0.05, and **P < 0.01 [34]

Recent research suggests another possible mode of action of yucca in preventing arthritis by anti-inflammatory activity. Yucca contains anti-inflammatory polyphenolics such as resveratrol and yuccaols A, B, C, D and E [18,19]. Yucca bark and whole yucca plant powder contain resveratrol (Table 1), well known for its anti-inflammatory activity [20,33]. Marzocco [34] demonstrated that yuccaols inhibit inducible nitric oxide synthase (iNOS) expression (Fig. 3). Nitric oxide is an inflammatory agent, and its content in tissues increases during inflammatory responses. The expression of iNOS is controlled by NFkappaB (NFkB), a transcription factor that regulates gene expression. Resveratrol and yucca phenolics strongly inhibit NFkB [34]. Yuccaol C is particularly effective (Fig. 3 and 4). Thus, whole plant yucca powder has powerful anti-inflammatory activity, mediated via inhibition of NFkB activation.

The generation of reactive oxygen species (free radicals) is an important factor in the development and maintenance of rheumatoid arthritis in humans and animal models [35]. One source of free radicals is nitric oxide produced within the synoviocytes and chondrocytes, giving rise to the highly toxic radical peroxynitrite [35]. The study of experimental arthritis in animals has demonstrated an increased activity of iNOS [36,37]. Thus the NFkB inhibitory and anti-oxidant effects of yucca polyphenolics may aid in prevention of reactive oxygen species (ROS) induction of arthritis by inhibiting the induction of iNOS.

Platelet aggregation is characteristic of inflammation. Yucca phenolics have inhibitory activity against platelet aggregation [38-40]. Yucca phenolics also have antioxidant activity [19] and free-radical scavenging effects [18]. Blood platelets participate in allergic inflammation responses [41]. Yuccaols inhibit the generation of free radicals in blood platelets [39]. One of the yucca phenolics, *trans*-3,3',5,5'-tetrahydroxy-4-methoxystilbene, showed the highest anti-platelet action.

Another botanical product with anti-inflammatory activity is cat's claw [42]. As reviewed by Miller et al. [42], cat's claw (*Uncaria guianensis*) "is a remarkably potent inhibitor of NFkB activity and tumor necrosis factor production." Evaluation of the anti-inflammatory activity of a combination of yucca and cat's claw would be of interest.

The evidence presented in this review indicates that yucca has potential in vivo anti-inflammatory activity, and warrants more in-depth investigation.

## Conclusion

*Yucca schidigera *is a medicinal plant which may have beneficial effects in the prevention and treatment of arthritis. Active components of yucca include steroidal saponins and polyphenolics such as resveratrol and yuccaols. Saponins may have anti-arthritic effects associated with their anti-protozoal activity. Yucca polyphenolics may have several roles in anti-arthritic activity. They inhibit NFkB, a transcription factor which stimulates iNOS, an inducible enzyme which produces the inflammatory agent nitric oxide. Yucca phenolics also are antioxidants and free-radical scavengers, which may aid in suppressing reactive oxygen species (ROS) that stimulate inflammatory responses. Folk medicine and anecdotal reports suggest that whole yucca plant powder aids in prevention and treatment of arthritis. Further studies on the anti-arthritic effects of yucca are warranted.

## Competing interests

PRC is a consultant to Desert King International (DKI), a privately-held company which produces and markets yucca extracts and yucca powder as commodities. He has no equity interest in this company. SP and WO have no relationships with DKI.

## Authors' contributions

PRC wrote the paper.

SP provided Figures 3 and 4, and the discussion in the paper associated with these figures.

WO provided Figures 1 and 2, and the discussion in the paper associated with these figures. He also developed the collaboration with SP, and collectively they demonstrated the role of yucca phenolics as inhibitors of NFkB and iNOS production.
